# Effect of Formulated *Artocarpus champeden* Extract on Parasite Growth and Immune Response of *Plasmodium berghei*-Infected Mice

**DOI:** 10.1155/2020/4678634

**Published:** 2020-02-28

**Authors:** A. Widyawaruyanti, N. Harwiningtias, L. Tumewu, A. F. Hafid, Soetjipto

**Affiliations:** ^1^Department of Pharmacognosy and Phytochemistry, Faculty of Pharmacy, Universitas Airlangga, Surabaya 60115, Indonesia; ^2^Natural Product Medicine Research and Development, Institute of Tropical Disease, Universitas Airlangga, Surabaya 60115, Indonesia; ^3^Faculty of Pharmacy, Universitas Airlangga, Surabaya 60115, Indonesia; ^4^Department of Biochemistry, Faculty of Medicine, Universitas Airlangga, Surabaya 60132, Indonesia

## Abstract

**Background:**

The ethanol extract of *Artocarpus champeden* stem bark (ACEE) has been proven to exhibit antimalarial activity. Despite the antimalarial effects observed, mechanisms of immune response to explain the antimalarial activity of ACEE remain poorly characterized. Here, we show the production of pro- and anti-inflammatory cytokines T helper 1 (Th1: IFN-*γ*, TNF-*α*) and T helper 2 (Th2: IL-10) from *Plasmodium berghei*-infected mice treated with formulated ACEE in order to better characterize the mechanism behind ACEE's antimalarial activity. In addition, we have also determined the effect of formulated ACEE on parasite growth and liver function.

**Methods:**

Balb/c mice were infected with *P. berghei* strain ANKA and then administered daily doses of ACEE at a dose of 20, 50, and 100 mg/kg BW, and survival time was recorded. We determined the presence of *P. berghei* strain ANKA and then administered daily doses of ACEE at a dose of 20, 50, and 100 mg/kg BW, and survival time was recorded. We determined the presence of *P. berghei* strain ANKA and then administered daily doses of ACEE at a dose of 20, 50, and 100 mg/kg BW, and survival time was recorded. We determined the presence of *γ*, TNF-*α*) and T helper 2 (Th2: IL-10) from

**Results:**

We found that formulated ACEE inhibited parasite growth and showed the highest antimalarial activity at 100 mg/kg BW. AST and ALT levels were found to be in the normal range, and there was no significant difference among control and treatment groups (*P* > 0.05). Infected mice treated with formulated ACEE showed a significant increase in the production of IFN-*γ*, TNF-*α*) and T helper 2 (Th2: IL-10) from

**Conclusion:**

This study suggests that the administration of ACEE was effective in inhibiting *P. berghei* strain ANKA and then administered daily doses of ACEE at a dose of 20, 50, and 100 mg/kg BW, and survival time was recorded. We determined the presence of *γ*, TNF-*α*) and T helper 2 (Th2: IL-10) from

## 1. Introduction

In 2018, there were an estimated 228 million new cases of malaria worldwide, in which 405,000 were estimated to have resulted in death [1]. Between 2010 and 2015, the malaria mortality rate among children under five years of age fell by an estimated 35%. Nevertheless, malaria remains a significant threat to under-fives, claiming the life of one child every two minutes [1]. Moreover, increasing resistance to current antimalarial drugs remains a threat to global efforts to control and eliminate malaria [2]. Therefore, safe and effective alternative antimalarial drugs are urgently needed.

Plants have long been potential sources of antimalarial drugs. *Cinchona* species are well known for their antimalarial properties. Quinine, isolated from *Cinchona* species, is one of the most common drugs used to treat malaria. *Artemisia annua* has been used in traditional Chinese medicine, and artemisinin, its active compound, has a higher chemotherapeutic index than chloroquine, a common antimalarial drug [3]. Plants used to treat malaria traditionally are potential sources of new antimalarial drugs [4, 5].

Indonesia's high biodiversity makes it an excellent bioprospecting source for new antimalarial drugs. *Artocarpus champeden* (Moraceae family), which in Indonesia is commonly known as *cempedak*, has been used as a traditional medicine to cure malaria [6]. In our previous studies, several prenylated flavones were isolated from *A. champeden* stem bark extract and showed potent antimalarial activity against *Plasmodium falciparum* [7]. Heteroflavanone C was the most potent compound among the compounds isolated, which has inhibitory activity against the growth of *P. falciparum* 3D7 strain with an IC_50_ value of 0.0006 *µ*g/mL [7]. Furthermore, artopeden A, an isoprenylated flavone, was also isolated from the bark of *A. champeden*. This compound showed potent antimalarial activity with an IC_50_ value of 0.045 *μ*g/mL [8]. Hafid et al. isolated another antimalarial active compound from *A. champeden* stem bark, which identified as morachalcone A with an IC_50_ value of 0.18 *μ*g/mL [9].

It is known that the most substantial content of *A. champeden* is flavonoids [10]. Flavonoids can affect immune responses and act as an anti-inflammatory agent. This has been demonstrated in vitro by inhibiting TNF-*α* (proinflammatory cytokines) as well as through upregulation of Th1 and downregulation mechanisms such as Th2 [11–13]. Another study has been done by Chang (2007), who stated that flavonoids modulate IFN-*γ* expression [14]. Flavonoids also have the ability to increase the production of NO and tumor necrosis factor (TNF-*α*) [15]. Flavonoids isolated from *Bidens pilosa*, centaurin, and centaureidin can increase IFN-*γ* production in T cells of the mice spleen [14]. Therefore, we assumed that flavonoids from *A. champeden* were possible to have activity in modulating TNF-*α* and IFN-*γ* expression as well.

In malaria infection, cytokines are mediators involved in pathogenesis and the elimination of the parasite [16]. Clark stated that TNF-*α* is a mediator of severe malaria. Proinflammatory cytokines, including TNF-*α* and IFN-*γ*, are thought to be responsible for cerebral symptoms [16, 17]. Cytokines control *Plasmodium* infection intracellularly through Th1 cells. Later, this requires counterbalance through the Th2 response to eliminate the pathogen extracellularly by Th2 cells by producing antibodies. IL-10 is a cytokine of Th2 that can decrease parasitemia in the late phase [18].

In order to develop the herbal product of *A. champeden*, its stem bark extract was further formulated by adding several excipients as capsule dosage form (formulated ACEE: *Artocarpus champeden* ethanol extract). The formulation of the extract could affect the activity; therefore, the antimalarial activity of the formulated extract needed to be evaluated. In this study, there is evidence that the administration of formulated ACEE was effective in inhibiting *P. berghei* growth in infected mice and extending survival time. No effect on liver function was observed based on AST and ALT levels. Currently, the immune response to formulated ACEE administration is not well known, especially in relation to malaria. Herein, we demonstrated the effect of formulated ACEE on host immune response, especially on the expression of Th1/Th2 cytokine when infected by *P. berghei*. Treatment of formulated ACEE resulted in an increased production of cytokines Th1 (IFN-*γ*, TNF-*α*) when compared with untreated mice.

## 2. Materials and Methods

### 2.1. Material

An 80% ethanol extract of *A. champeden* stem bark (ACEE) was formulated as a capsule dosage form containing 15 mg extract per 300 mg granule. A wet granulation method was conducted to produce formulated ACEE by adding several excipients, including PEG 6000, PVP K25, lactose, Avicel PH-101, and Primogel. The formula is proprietary. The formulation was produced at the Faculty of Pharmacy, Universitas Airlangga, Surabaya, Indonesia.

### 2.2. Parasite

*P. berghei* ANKA strain was obtained from the Eijkman Institute for Molecular Biology, Jakarta. This parasite has been maintained at the Institute of Tropical Disease, Universitas Airlangga, Surabaya, Indonesia.

### 2.3. Animals

Female mice Balb/c strain at the age of 6–8 weeks and 20–30 g of weight was obtained from the Animal Experimental Development Unit–Gadjah Mada University (Yogyakarta, Indonesia). Animals were maintained at the Animal Laboratory of the Institute of Tropical Disease, Universitas Airlangga. Permission and approval for animal studies were obtained from the Faculty of Veterinary Medicine, Universitas Airlangga, with ethical clearance No: 194-KE.

### 2.4. *In Vivo* Antimalarial Activity and Effect on Liver Function

*In vivo* antimalarial testing was performed based on Peter's test (a 4-day suppressive test) with modifications [19]. The test was performed using 6 mice per group. Mice were infected intraperitoneally with 0.2 mL (1 × 10^5^) of *P. berghei* on day 0. The treatment was initiated after parasite infection occurred (day 2). *P. berghei-*infected mice were treated orally with formulated ACEE (granule form suspended in CMC-Na 0.5%) twice a day for five days (day 2 until day 6) at a dose equal to ACEE 20, 50, and 100 mg/kg body weight. Giemsa-stained thin blood smears were made for 7 days (day 2 until day 8). The infected red blood cells were counted under the microscope to determine the parasitemia and the inhibition of parasite growth. Mice blood was taken intracardially at day 8 to determine the effect on liver function by the observed AST and ALT levels.

The percentage of growth inhibition of *P. falciparum* was calculated using the following formula:

(1) $$\begin{array}{c} 100-\left(\frac{X_{e}}{X_{k}}\times 100\%\right), \end{array}$$

where *X*_*e*_ is the % parasitemia growth of an experimental group and *X*_*k*_ is the % parasitemia growth of the negative control.

### 2.5. Determination of Immune Response (IFN-*γ*, TNF-*α*, and IL-10) on *P. berghei*-Infected Mice

Female mice Balb/c strain was divided into uninfected and *P. berghei*-infected groups. Each group was further divided into untreated and treated groups. Each group consisted of 6 mice. For the infected group, mice were infected intraperitoneally with 0.2 mL (1 × 10^5^) of *P. berghei* on day 0. For the treated group, mice were given ACEE orally (granule form suspended in CMC-Na 0.5%) at a dose of 100 mg/kg BW or CMC-Na 0.5% twice a day for four days (day 2 until day 5). ACEE at a dose of 100 mg/kg BW was chosen based on previous *in vivo* antimalarial test results (Table 1). The uninfected and untreated group was used as controls to determine the normal levels of IFN-*γ*, TNF-*α*, and IL-10. The infected and untreated group was used as a natural malaria pathogenesis model. Parasitemia was observed every day (day 2 until day 12). On days 2, 4 and 7 after infection, mice were euthanized, and blood was collected to determine IFN-*γ*, TNF-*α*, and IL-10 by ELISA. Indirect sandwich ELISA TNF-*α*, IFN-*γ*, IL-10 cytokine of TNF-*α*, IFN-*γ*, and IL-10 was conducted using pairs of capture and detection Abs (BioLegend: Mouse cytokine ELISA MAXTM Deluxe Sets).

### 2.6. Statistical Analysis

The numerical data are presented as mean ± standard deviation. Data were analyzed by SPSS 20.0. The significance of the mean difference between independent groups was determined by using a one-way analysis of variance (ANOVA) and least significant difference (LSD) analysis. The *P* value <0.05 is considered significant.

## 3. Result

### 3.1. In Vivo Antimalarial Activity and Toxicity Effect on Liver Function

**Figure 1:**
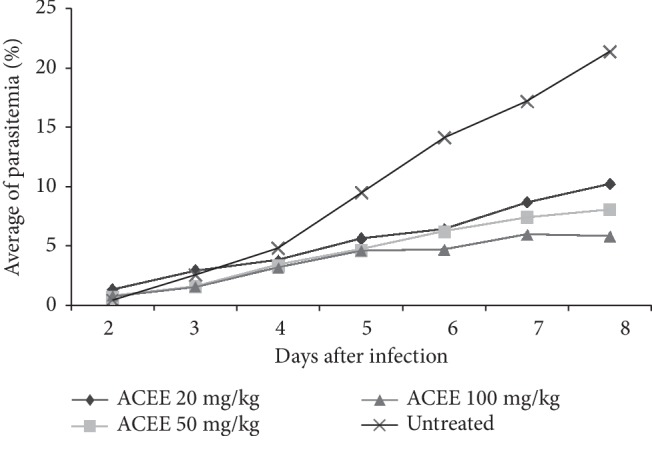
The mean of parasitemia of treated and untreated groups on day 2 until day 8 after parasite infection.

Formulated ACEE at a dose equal to ACEE 20, 50, and 100 mg/kg inhibited parasite growth by 57.36% (±9.18), 64.48% (±5.38), and 72.24% (±4.30), respectively. The highest inhibition shown was at a dose equal to ACEE 100 mg/kg. There was a significant difference in parasite growth inhibition among groups (*P* > 0.05). The parasite growth and inhibition data are described in Table 1 and Figure 1.

The effect of ACEE on liver function was observed by determining the AST and ALT levels of the treated mice groups compared with the control group. The AST and ALT levels of the treated groups ranged from 264.33–278.33 U/L and 79.16–115.66 U/L, respectively. Meanwhile, AST and ALT levels of the control group were 266.28 U/L and 89.57 U/L, respectively.

The normal AST and ALT levels of mice are 70–400 U/L and 25–200 U/L, respectively. We observed that AST and ALT of all groups were in the normal range. Statistical analysis results showed that there were no significant differences in AST and ALT levels among groups (*P* > 0.05). The AST and ALT levels are described in Table 2.

ACEE showed the highest antimalarial activity at a dose equal to ACEE 100 mg/kg; no effect on liver function based on AST and ALT levels was observed. Therefore, further investigation of the immune response of *P. berghei*-infected mice treated with ACEE was done by administering ACEE at a dose equal to 100 mg/kg.

### 3.2. Determination of Immune Response (IFN-*γ*, TNF-*α*, and IL-10) on *P. berghei*-Infected Mice

#### 3.2.1. Parasitemia Observation

The antimalarial activity of ACEE on *P. berghei*-infected mice was determined by the percentage of parasitemia (observation on day 2 until day 12), parasitemia growth, and inhibition of growth (observation on day 4, day 7, and day 12). The results showed that on the infected and untreated groups, the parasites began to grow on day 2 and increased until day 12. ACEE demonstrated the highest inhibitory effect on parasite growth on day 4 (Table 3).

#### 3.2.2. Survival Rate Observation

There was increased survival for infected mice given ACEE, in which mean of survival days was 11.83 (±1.60) days. Infected and untreated mice survived until day 11. Meanwhile, the administration of CMC-Na 0.5% resulted in decreased mean survival days by 10.33 (±1.75) days. It was possibly caused by a high parasitemia level of mice in infected/untreated and infected/CMC-Na groups (Table 3).

#### 3.2.3. Cytokine Determination

Immune responses of mice were conducted through the determination of the production of cytokine Th-1 (IFN-*γ* and TNF-*α*) and cytokine Th-2 (IL-10) (Table 4).

Statistical analysis showed that there was no significant difference in cytokine production between uninfected groups on day 4 and day 7. However, the production of TNF-*α* was significantly different between the infected/ACEE group and the other groups on day 4. There was no significant difference between the infected/ACEE group and the infected/CMC-Na group on day 7. The production of IFN-*γ* was significantly different between uninfected and infected groups on day 4 and day 7. Infected groups had increased TNF-*α* and IFN-*γ* levels compared with uninfected groups. IL-10 production was not different among groups on day 4 and day 7.

## 4. Discussion

### 4.1. In Vivo Antimalarial Activity and Toxicity Effect on Liver Function

*Artocarpus* (Moraceae family) contains approximately 50 species; many of these are used in traditional medicine [20]. Several *Artocarpus* species are reported to exhibit antimalarial activities against *P. falciparum* and *P. berghei*. A prenylated stilbene compound isolated from aerial parts of *A. integer* was reported to have antimalarial activity with an IC_50_ value of 1.7 *μ*g/mL [21]. *A. altilis* leaf extract showed antimalarial activity with an IC_50_ value of 1.32 *μ*g/mL against *P. falciparum* and ED_50_ value of 0.82 mg/kg body weight against *P. berghei* [22]. Several compounds isolated from *A. champeden* extract showed outstanding activity against *P. falciparum* [7–9]. Therefore, *A. champeden* stem bark extract was formulated as a capsule dosage form (formulated ACEE) and further developed as an antimalarial drug.

This study showed that formulated ACEE exhibited antimalarial activity against *P. berghei* in a dose-dependent manner. Formulated ACEE at a dose equal to ACEE 20, 50, and 100 mg/kg body weight given twice a day for five days inhibited *P. berghei* growth by more than 50% by day 8 of observation.

Antiplasmodial activity of an extract that performs percentage parasitemia suppression ≥50% at a dose of 500, 250, and 100 mg/kg body weight per day in vivo can be classified as moderate, good, and very good, respectively [23]. Based on this classification, we can suggest that ACEE showed very good activity at doses of 20, 50, and 100 mg/kg body weight. The highest inhibition resulted from ACEE at a dose of 100 mg/kg body weight. This result was in accordance with previous study as stated above [7–9, 21, 22].

The results of liver function observation showed that AST and ALT levels of treated groups and the untreated group were in a normal range. It is therefore likely that ACEE treatment, even at the highest dose, does not affect the liver function.

### 4.2. Determination of Immune Response (IFN-*γ*, TNF-*α*, and IL-10) on *P. berghei*-Infected Mice

The results showed that TNF-*α* levels were higher with lower parasitemia in the infected/ACEE-treated group compared with infected/CMC-Na-treated and infected/untreated group on day 4 and day 7. An increased TNF-*α* level was in accordance with decreased parasitemia in the infected group. ACEE treatment increased cytokine production that resulted in lower parasitemia. This is shown by lower parasitemia in the infected/ACEE group compared with infected/CMC-Na and infected/untreated group on day 4 and day 7. Increased TNF-*α* at the beginning of the infection is likely to have inhibited parasite multiplication and provided immunity protection through plasmodium [24, 25]. TNF-*α* is induced in monocytes and macrophages when infected erythrocytes rupture, releasing parasite antigens into the bloodstream. TNF-*α* also acts on lymphocytes and monocytes by increasing *P. falciparum* inhibition via mechanisms without phagocytosis [26]. Previous studies have reported that TNF-*α* increases within 24 hours after *P. berghei* infection. The response of TNF-*α* at the beginning of the infection indicates immunity protection against plasmodium [25]. Macrophage activation as an initial response after the infection was likely to have increased TNF-*α* production and stimulate NK cells to produce IFN-*γ* [27].

IFN-*γ* levels were significantly increased on day 7, and the infected/ACEE group had the highest IFN-*γ* of 290.32 ± 100.64 pg/mL compared with infected/untreated and infected/CMC-Na groups with IFN-*γ* of 185.22 ± 33.27 pg/mL and 225.13 ± 61.13 pg/mL, respectively. IFN-*γ* is a Th1 cytokine, which activates macrophage. Active macrophage will release NO to eliminate the parasites present [28]. This condition was in accordance with the result in which the IFN-*γ* level was higher in the group with lower parasitemia (infected/ACEE) compared with the group with higher parasitemia (infected/untreated and infected/CMC-Na) on day 4 and day 7.

The TNF-*α* and IFN-*γ* levels of the infected/ACEE group were increased on day 7, even though inhibition of parasite growth considered high compared with the infected/untreated group. Prolonged induction of proinflammatory cytokines might be associated with the risk of developing severe disease. Therefore, IL-10 was necessary for proinflammatory suppression in the host during infection [29]. Meanwhile, this study result showed that the production of IL-10 on infected groups was not significantly different among groups on day 4 and day 7. ACEE treatment has not demonstrated the ability to balance the Th1 (TNF-*α*, IFN-*γ*) and Th2 (IL-10) response. Additional study is needed to improve antimalarial outcomes of ACEE treatment. Observation of the toxic effect regarding the modulating impact of proinflammatory cytokines needs to be addressed.

The IFN-*γ* levels in the uninfected/ACEE group were higher than those in the uninfected/CMC-Na group on day 7. This result demonstrates that ACEE could stimulate IFN-*γ* production in normal conditions. Therefore, it is possible to use ACEE for preventative malaria treatment.

## 5. Conclusions

This study suggests that the administration of formulated ACEE was effective in inhibiting parasite growth in *P. berghei*-infected mice and there was no effect on liver function based on AST and ALT levels. Furthermore, ACEE treatment was associated with the enhanced production of the proinflammatory cytokines IFN-*γ* and TNF-*α*. ACEE treatment also decreased the parasitemia level and extended survival time of *P. berghei*-infected mice. The formulated *Artocarpus champeden* ethanol extract (ACEE) treatment may provide novel therapeutic strategies for malaria.

## Figures and Tables

**Table 1:** Percentage of parasite growth and inhibition of *P. berghei* at day 6 and day 8.

Group	Parasite growth (%)^*∗*^		Parasite growth inhibition (%)^*∗*^	
	Day 6	Day 8	Day 6	Day 8
ACEE 20 mg/kg	1.28 ± 0.26	1.48 ± 0.32^a^	62.67 ± 7.49	57.36 ± 9.18^a^

ACEE 50 mg/kg	1.39 ± 0.24	1.24 ± 0.18^b^	59.34 ± 6.98	64.48 ± 5.38^a^

ACEE 100 mg/kg	1.00 ± 0.26	0.97 ± 0.15^a^	70.66 ± 7.58	72.24 ± 4.30^a^

Untreated	3.42 ± 0.61	3.48 ± 0.33^ab^	—	—

^*∗*^Data represent mean ± SD (*n* = 6); ^a,b^significant difference among groups (*P* < 0.05); untreated group as control group.

**Table 2:** AST and ALT levels of the treated and control groups.

Group	AST (U/L)^*∗*^	ALT (U/L)^*∗*^
ACEE 20 mg/kg	278.33 ± 31.05	85.66 ± 29.14
ACEE 50 mg/kg	264.33 ± 25.53	115.66 ± 34.91
ACEE 100 mg/kg	269.00 ± 70.01	79.16 ± 17.87
Untreated	266.28 ± 67.48	89.57 ± 40.22

^*∗*^Data represent mean ± SD (*n* = 6); no significant different among groups (*P* > 0.05).

**Table 3:** Parasite growth and inhibition on *P. berghei*-infected mice.

Groups	Parasitemia growth (%)^*∗*^			Inhibition (%)^*∗*^			Mean of survival days
	Day 4	Day 7	Day 12	Day 4	Day 7	Day 12	
Infected/untreated	1.38 ± 0.08^a^	4.55 ± 0.92^c^	4.48 ± 0.29^d^	—	—	—	11.00 ± 0.00
Infected/CMC-Na	1.37 ± 0.14^b^	6.10 ± 1.87^c^	5.92 ± 1.77^d^	NI	NI	NI	10.33 ± 1.75
Infected/ACEE	0.36 ± 0.22^ab^	1.91 ± 0.57^c^	3.75 ± 0.89^d^	74.03 ± 16.16	55.53 ± 12.65	36.53 ± 15.09	11.83 ± 1.60

^*∗*^Data represent mean ± SD (*n* = 6); infected/untreated group as control group; NI: no inhibition; ^a,b,c,d^significant difference among groups (*P* < 0.05).

**Table 4:** Cytokines productions (IFN-*γ*, TNF-*α* and IL-10) of mice.

Groups	TNF-*α* (pg/mL)			IFN-*γ* (pg/mL)			IL-10 (pg/mL)		
	Day 2	Day 4	Day 7	Day 2	Day 4	Day 7	Day 2	Day 4	Day 7
Uninfected/CMC-Na	0.04 ± 0.00	1.46 ± 0.76^ab^	1.21 ± 0.62^ab^	18.09 ± 6.97	18.74 ± 0.29^abc^	18.56 ± 0.00^abc^	49.76 ± 8.30	46.19 ± 2.15	45.24 ± 1.73
Uninfected/ACEE	0.04 ± 0.00	1.12 ± 0.48^cd^	0.66 ± 0.19^cd^	18.09 ± 6.97	19.4 ± 2.01^def^	73.09 ± 38.32^def^	49.76 ± 8.30	45.95 ± 2.29	46.67 ± 2.15
Infected/untreated	1.34 ± 0.50	3.39 ± 1.25^ace^	3.77 ± 2.59^abcd^	210.50 ± 96.50	256.52 ± 56.57^ad^	185.22 ± 33.27^ad^	74.52 ± 48.15	51.19 ± 13.44	77.14 ± 50.32
Infected/CMC-Na	1.34 ± 0.50	3.74 ± 0.84^bd^	7.94 ± 3.30^ac^	210.50 ± 96.50	189.11 ± 103.99^be^	225.130 ± 61.13^be^	74.52 ± 48.15	60.00 ± 19.65	49.52 ± 3.69
Infected/ACEE	1.34 ± 0.50	5.99 ± 2.08^abcde^	9.19 ± 3.58^bd^	210.50 ± 96.50	208.74 ± 78.39^cf^	290.32 ± 100.64^cf^	74.52 ± 48.15	51.67 ± 12.69	51.19 ± 11.55

^*∗*^Data represent mean ± SD (*n* = 6); ^a,b,c,d,e,f^significant difference among groups (*P* < 0.05).

## Data Availability

The data used to support the findings of this study are included within the article.
